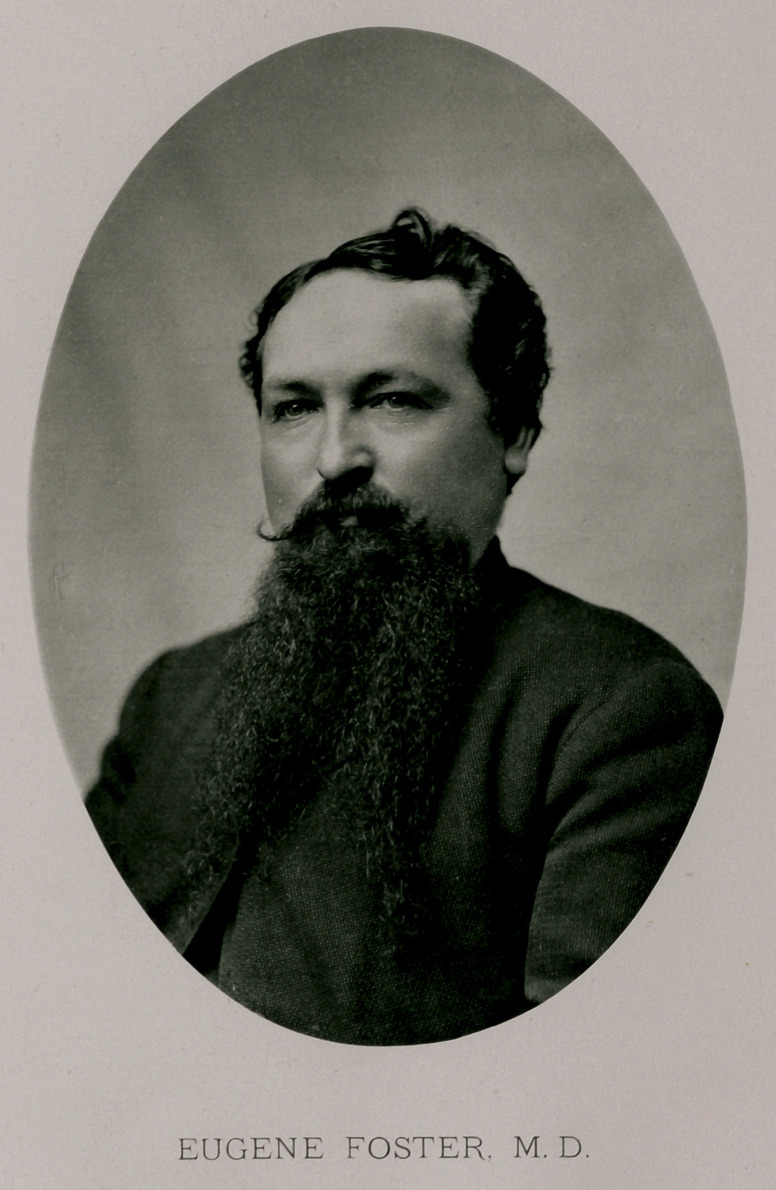# Our Portrait Gallery

**Published:** 1884-10

**Authors:** 


### OUR PORTRAIT GALLERY.

### EUGENE FOSTER, M. D.

Dr. Eugene Foster, the subject of this sketch, was born in Augusta, Georgia, April 7th, 1850. His father was the Hon. John Foster, of that city, one of the most popular and worthy citizens of the State. Having received an academic education, he began the study of medicine in the fall of 1868, and was graduated M.D. from the Medical College of Georgia (now the Medical Department of the State University), on the 1st of March, 1872. The remainder of that year was spent by him in attendance upon college clinics and hospitals, where he enjoyed extraordinary opportunities for advancement in a knowledge of his chosen profession. Returning from New York in the winter of 1872, he entered upon the practice of medicine in all of its branches, taking, at once, a high position as a practitioner.

EUGENE FOSTER, M. D.

In 1873 Dr. Foster was appointed physician in charge of the small-pox Hospitals in Augusta, in which position he was charged with the duty of arresting the spread of that epidemic which so seriously threatened the city. In this he was eminently successful. In 1874 he held a similar position with like success. During these years he was physician in charge of the small-pox hospitals of Richmond county, and held the epidemic thoroughly under his control.

In 1876, when the city of Augusta was seriously threatened by yellow fever, he was appointed health officer, and as such was entrusted with the entire management of the quarantine and inspection services which were enforced there and on the railroad trains entering therein.

In 1880 he was appointed a member of the Board of Health of Augusta, and unanimously chosen president of that body, succeeding to the unexpired term of Dr. L. D. Ford. He was unanimously re-elected in 1881 and 1884—his term of office continuing until May, 1888. On the occasion of his second re-election to the presidency of the Board of Health, the members of that body, as a testimonial of their personal regard and their high appreciation of his eminent services to his native city, presented him with an elegant gold watch and chain and seal on which were engraved the sentiments of the donors.

Among the professional papers contributed by him are the following; "Carbolic Acid as Local Anæsthetic in Surgical Operations;" "Treatment of Constitutional Syphilis;" "History of Epidemics of Yellow Fever in Augusta, Georgia;" "The Most Effectual Means of Preventing and Controlling Small-Pox;" "Sanitary Condition and Needs of Augusta;" "Examination of Alleged Dangers to Health from Excavations of Earth in Spring and Summer Seasons;" "Sanitation—Its Importance and Economy;" "Prevention and Control of Small Pox by Vaccination, Isolation and Disinfection;" "The Relative Merits of Humanized and Bovine Vaccine Virus;" "Compulsory Vaccination—Laws of England, Ireland, Scotland, Germany and France, with Considerations as to the Probable Results of such a Law applied to America;"

"Municipal Organization of the American Public Health Service;" "Syphilitic Diseases of the Brain;" "Diagnosis and Treatment of Small-Pox;" "Dengue Fever;" "Syphilis as a Sociological Problem; the Opinions and Statements of Herbert Spencer thereon Reviewed;" "The Sewerage and Drainage of Augusta, Georgia;" "The Water Supply of Augusta, Georgia;" "Stricture of Urethra;" "Treatment of Phimosis by Dilatation, etc."

For the last ten years he has been a member of the Medical Association of Georgia, manifesting a deep interest in its career of usefulness by a regularity of attendance upon its sessions and his efforts to build up the Association. He has served as chairman of the committee on Inebriate Asylums; of the committee on Prize Essays, and of the committee on Necrology, and as a member of the Board of Censors. He has also served as vice-president of the Association, and in 1884 was chosen its president, showing his high standing in the estimation of the members of this distinguished body of physicians.

Dr. Foster is a member of the American Public Health Association, and also of the American Medical Association, in both of which he holds positions on their most important committees, and has contributed to both interesting and valuable medical papers.

This brief and imperfect outline of his professional career clearly indicates his devotion to his chosen profession and his constant efforts to advance its usefulness. His life has been remarkable for the labor he has performed in endeavors to acquaint himself thoroughly with the science of medicine and to make new discoveries in the nature, prevention and cure of maladies which affect the health and life of his fellow-man. It is safe to predict great achievements for one so assiduous in his efforts—who regards idleness as a vice, and who esteems it his chief delight to glean from the field of Medical Science. He does not belong to that class who consider everything accomplished, or who are content to receive, as their full meed of knowledge, the acquirements of others. He lives and acts under the conviction that there is yet unattained knowledge, and that it is his duty as well as his right to discover, by research and experiment, hidden facts of science.

It is also manifest from the positions he has held and still holds, that he enjoys the highest confidence and esteem of his professional brethren.

With all he is a gentleman of true modesty, never failing to recognize as his equal every man who honestly labors to perform his entire duty. With a heart full of noble and generous impulses, he wins friends wherever he goes, the poor and needy never turning to him for help in vain. To his scientific attainments he has added the accomplishments of literary culture, while his genial nature renders him a favorite in the high social circle in which he moves. Still young and in vigorous health, there is before him a prospective career of usefulness and distinction which may well be envied. Already his life has blessed mankind, and is an exemplar worthy of imitation.

## Figures and Tables

**Figure f1:**